# Predictors of orthorexic behaviours in patients with eating disorders: a preliminary study

**DOI:** 10.1186/s12888-015-0628-1

**Published:** 2015-10-15

**Authors:** Anna Brytek-Matera, Radosław Rogoza, Carla Gramaglia, Patrizia Zeppegno

**Affiliations:** SWPS University of Social Sciences and Humanities, Faculty in Katowice, Techników 9, 40-326 Katowice, Poland; University of Cardinal Stefan Wyszynski, Wóycickiego 1/3b, 01-938 Warsaw, Poland; Dipartimento di Medicina Traslazionale, Institute of Psychiatry, Università degli Studi del Piemonte Orientale, C.so Mazzini 18, 28100 Novara, Italy

**Keywords:** Eating disorders, Orthorexia, Risk factors

## Abstract

**Background:**

The construct of orthorexia in eating disorders (EDs) has received very little attention despite clinical observations of a possible overlap between the two. The aim of this study was: 1) to assess orthorexic behaviours, eating disorder pathology and attitudinal body image in ED patients; 2) to identify possible predictors of orthorexia nervosa among ED patients.

**Methods:**

Fifty-two women diagnosed with EDs were recruited. Patients’ assessment included the following: the ORTO-15 test (Polish version) for orthorexic behaviours; the Eating Attitude Test-26 (EAT-26) to identify ED symptoms; the Multidimensional Body-Self Relations Questionnaire (Polish version) to assess body image.

**Results:**

A latent class analysis was performed and differences between identified classes were assessed. The main differences concerned weight, ED pathology and orthorexic behaviours within the same group of ED patients. In order to examine predictors of orthorexia nervosa, we investigated a structural equation model, which excellently fitted to the data (*χ*^*2*^_(17)_ = 23.05; *p* = .148; CFI = .962; RMSEA = .08; *p* = .25; SRMR = .05). In ED patients, orthorexic behaviour was negatively predicted by eating pathology, weight concern, health orientation and appearance orientation.

**Conclusion:**

The assessment of the orthorexia construct in EDs may add to the paucity of studies about this issue and may help to clarify the relationship between the two. Differences and similarities seem to exist between these disorders, and may benefit from specific treatment approaches. Moreover, these preliminary findings open tracks for future research in the field of the psychology of eating.

## Background

The word “orthorexia” literally means “proper appetite”, and the phrase “orthorexia nervosa” was suggested by Bratman [1] to indicate the pursuit for healthy food consumption. Although the phrase orthorexia nervosa should be applied only to pathological conditions, it is commonly used to describe states ranging from healthy behaviours to a pathological interest for healthy and pure food [2]. Even if socially acceptable and laudable to a certain extent, orthorexic behaviours can possibly become pathological when leading to an exclusive focus of one’s life on a proper diet, with a consequent impairment of functioning (e.g., social, relational…) and of health. As far as this last point is concerned, the literature is not univocal; some Authors claim that orthorexia is more a source of psychological than physical distress, while others think it can lead to consequences similar to those of anorexia nervosa [3–6].

Orthorexia is supposed to share features and possibly overlap with other psychiatric disorders, including anorexia nervosa, obsessive-compulsive disorder, obsessive-compulsive personality disorder, somatic symptom disorder, illness anxiety disorder, and psychotic spectrum disorders [3]. Regarding the overlap between orthorexia and EDs, they share a lack of pleasure as far as eating is concerned, and a displacement of control over one’s own life onto food. Anyway, while orthorexic individuals focus on quality and purity of food, ED patients focus on food quantity. For the first the body needs to be pure, while for the latter the body needs to match an “ideal” of extreme thinness.

Apart from these clinical observations, the literature about the orthorexia construct in EDs is in its starting phase. To our knowledge, the number of studies investigating these issues is limited to the one by Segura-Garcia and coworkers [7], who found that orthorexia is frequently comorbid with EDs, including both anorexia and bulimia, and, interestingly, its frequency increases over time (from 28 to 53 % in a 3-year follow-up study). Thus, a further complication of the relationship between orthorexia and EDs is their possible coexistence or succession over time. Orthorexia may precede the onset of a full-syndrome ED, or, as suggested by Segura-Garcia and coworkers [7], it may represent its evolution during remission and recovery. From this point of view, a key issue of orthorexia is that it may make the outcast ED patient feel again an accepted part of society; it is not by accident that orthorexia has been described as “a disease disguised as a virtue” [1].

With the objective of adding to the paucity of studies about orthorexia in ED patients, the first aim of this study was to examine differences among patients with EDs as far as orthorexic behaviours, ED pathology and attitudinal body image are concerned. The second objective was to identify the possible predictors of orthorexic behaviours in ED patients.

## Methods

### Participants

The study was carried out at the Polish National Center for Eating Disorders. All outpatients referring to the Center from May 2014 to November 2014 with a diagnosis of either anorexia or bulimia nervosa were asked to participate in the study and, if available, were assessed in the starting phase of their treatment. Oral and written informed consent was obtained from all the patients. No further inclusion/exclusion criteria were applied. Diagnoses were made with the aid of the Structured Clinical Interview for DSM-IV-TR Axis I disorders [8]. Patients’ weight and height were recorded and body mass index (BMI) was calculated; participants were granted anonymity and were asked for informed consent. The study protocol has been approved by the SWPS University of Social Sciences and Humanities Human Research Ethics Committee.

Patients were asked five yes/no questions to gather data about weighing behaviours, body satisfaction and intentional weight loss; moreover, their assessment included the following measures:

### The Polish version of the ORTO-15 test

The original measure was developed by Donini and coworkers [9] to assess the so-called orthorexic behavior, which refers to the individuals’ obsessive attitude in choosing, buying, preparing and consuming food they consider healthy. Orthorexia nervosa is measured on a four-point Likert scale (from “always” to “never”), with items receiving a score = 1 reflecting an orthorexic tendency, and those with a score = 4 suggesting normal eating habits.

The ORTO-15 was validated in a Polish population [10], with a Cronbach’s alpha coefficient in the current study = 74. Only 9 items out of 15 determine the structure of the Polish version of the ORTO-15 test (*m* = 2.09; *SD* = 0.51); in the Polish version scores under the cut-off of 24 points indicate a strong preoccupation with consuming healthy food [11], while in the original measure the cut-off score is 40 points [12].

### The Polish version of the Multidimensional Body-Self Relations Questionnaire (MBSRQ)

The Polish version of the MBSRQ [11, 13] assesses the attitudes related to body image. The Polish version of the MBSRQ is composed of eight-factor subscales, differently from the original measures which includes 10 subscales [14]: (1) combined Appearance Evaluation and Body Areas Satisfaction scales, which assess feelings of attractiveness and body satisfaction (*m* = 2.68; *SD* = 0.61; *α* = .92); (2) Appearance Orientation scale, which measures the extent of investment in one’s appearance (*m* = 3.19; *SD* = 0.35; *α* = .86); (3) Fitness Evaluation scale, which evaluates feelings of being fit (*m* = 2.85; *SD* = 0.58; *α* = .85); (4) Fitness Orientation scale, measuring the extent of investment in being fit (*m* = 2.72; *SD* = 0.41; *α* = .75); (5) Health Evaluation scale, which assesses feelings of physical health (*m* = 3.12; *SD* = 0.47; *α* = .78); (6) Health Orientation scale, measuring the extent of investment in healthy lifestyle (*m* = 2.99; *SD* = 0.53; *α* = .76); (7) Self-Classified Weight scale which measures self-appraisals of weight (from very underweight to very overweight; *m* = 3.52; *SD* = 0.68; *α* = .47); and (8) Overweight preoccupation scale which measures fat anxiety, dieting and eating restraints (*m* = 3.70; *SD* = 1.02; *α* = .62).

### The Eating Attitude Test (EAT-26)

The EAT-26 [15] is a 26-item standardized self-report questionnaire investigating typical symptoms and attitudes of EDs in three areas: dieting, bulimia and food preoccupation, and oral control. The Dieting scale (*m* = 1.37; *SD* = 0.79) describes the pathological avoidance of fattening foods and body shape preoccupation. The Bulimia and Food Preoccupation scale (*m* = 1.03; *SD* = 0.93) evaluates bulimic behaviours and thoughts about food. The Oral Control scale (*m* = 0.56; *SD* = 0.58) explores the self-control about food and the social perceived social pressure to gain weight.

A score greater than 20 is considered to be an indicator of possible eating disordered behaviors and eating problems. The EAT-26 shows a satisfactory internal consistency [15]. Cronbach’s alpha coefficient for the total EAT-26 in this study was .72.

### Statistical analyses

For the first purpose of the study, a latent class analysis (LCA) was performed and differences between identified classes were assessed. All of the analyses were carried out in Mplus version 7.2 [16]. A structural equation model (SEM) was used for the second objective, i.e., to investigate predictors of orthorexic behaviours. Orthorexia, ED pathology, and body image issues were measured respectively by averaged total score of the ORTO-15 test, averaged factor scores of the EAT-26 test and of the MBSRQ. The estimation method we used was the robust Maximum Likelihood estimator with Satorra-Bentler corrections. To evaluate the model fit to the data we relied on likelihood ratio *χ*^*2*^ due to small sample size and additionally on approximate model fit indexes – the Comparative Fit Index (CFI) [17]; Root Mean Square Error of Approximation (RMSEA) with probability of close-fit hypothesis; and Standardized Root Mean Square Residual (SRMR). The model can be interpreted as well fitted to the data when *χ*^*2*^ and *p* value for close-fit hypothesis are insignificant, the CFI > .90, and RMSEA and SRMR < .08 [18, 19].

## Results

The response rate was 100 %; all the outpatients contacted during the study period agreed to take part in the research, for a total of 52 female patients with EDs. Patients’ mean age was 22.81 years (*SD* = 3.80). Mean BMI was 21.01 kg/m^2^ (*SD* = 2.53), which fits the standards for a normal weight (18.50–24.99 kg/m^2^) set by the World Health Organization (2000). The mean duration of eating-related problems was 1.85 years (*SD* = 0.36). The actual weight was 57.86 kg (*SD* = 9.81); the mean reported ideal weight was 50.17 kg (*SD* = 6.71).

The sample features are described in Table 1.

**Table 1:** Descriptive features of the sample (*N* = 52)

	*N* (%)
Diagnosis	
Anorexia nervosa	12 (23 %)
Bulimia nervosa	40 (77 %)
Nutritional status	
Severe thinness (BMI < 16)	1 (1.9 %)
Moderate thinness (16 < BMI < 16.99)	4 (7.7 %)
Mild thinness (17 < BMI < 18.49)	7 (13.5 %)
Normal weight (18.5 < BMI < 24.99)	40 (76.9 %)
Weighing oneself every day	
Yes	6 (11.5 %)
No	46 (88.5 %)
Body satisfaction	
Yes	8 (15.4 %)
No	44 (84.6 %)
Intentional weight loss^*^	
Dieting	26 (50.0 %)
Physical exercise	23 (44.2 %)
Using laxatives	7 (13.5 %)
Vomiting	10 (19.2 %)
Starvation	12 (23.1 %)

*For intentional weight loss, the numbers and % in the table refer to participants responding *yes*

82.7 % (43 our of 52) of patients presented a strong preoccupation with a healthy food intake based on the cut-off of 24 for the Polish version of the ORTO-15 test.

### Latent class analysis (LCA)

Differentiation of underlying latent classes was made according to statistical criteria. To do so we compared models from two to four latent classes; decision about number of latent classes was made on the basis of information criteria and Lo, Mendel, and Rubin [20] adjusted Likelihood Ratio test. Results of LCA are presented in Table 2.

**Table 2:** Fit statistics of latent class analysis for eating disorders patients

Number of classes	AIC	BIC	SSABIC	LRT	*p*
2	1037.52	1109.00	992.83	121.72	.05
3	1011.87	**1108.46**	951.48	50.66	.26
4	**1004.93**	1126.63	**928.84**	32.31	.85

Information criteria with smallest values are in bold character

*AIC* Akaike information criterion; *BIC* Bayesian information criterion; *SSABIC* sample size adjusted Bayesian information criterion; *LRT* Lo, Mendel, and Rubin (2001) adjusted Likelihood Ratio test

**Fig. 1:**
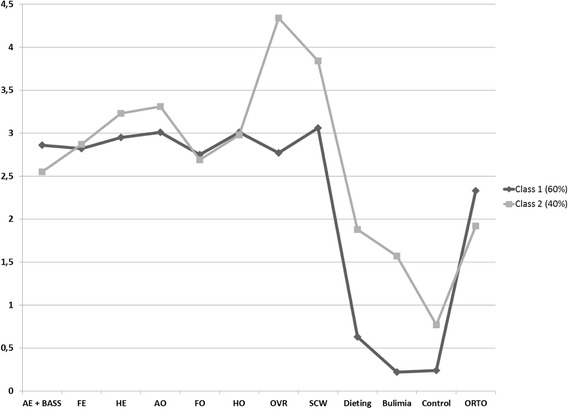
Latent profiles of eating disorders patients. Note. AE + BASS = Appearance Evaluation + Body Areas Satisfaction Scale; FE = Fitness Evaluation; HE = Health Evaluation; AO = Appearance Orientation; FO = Fitness Orientation; HO = Health Orientation; OVR = Overweight Preoccupation; SCW = Self-classified Weight

Despite the fact that Akaike and sample size adjusted Bayesian information criteria had smallest values and suggested to distinguish four latent classes, only the two classes solution was supported by Lo, Mendel, and Rubin test [20], which was found significant. Moreover the difference in BIC between two and three classes solution was marginal, therefore we decided to choose the two latent classes solution. Estimated mean scores for both groups are described in Fig. 1. It is worth noting that each questionnaire had different maximum averaged value (EAT: 3, ORTO: 4, and MBSRQ: 5), and difference of one point have different impact on different measures. Therefore the visual inspection of Fig. 1 should be made with caution and in accordance to maximum values of respective measures.

All differences were tested using Welch Test for heteroscedastic data. Differences between the two classes identified by the LCA were negligible and insignificant for Appearance Evaluation and Body Areas Satisfaction, Fitness Evaluation and Orientation, and Health Orientation. Differences were significant for Self-Classified Weight (*F*_(1, 28.82)_ = 18.60; *p* < .000), Overweight Preoccupation (*F*_(1, 37.55)_ = 65.86; *p* < .000), Dieting (*F*_(1, 43.06)_ = 82.44; *p* < .000), Bulimia (*F*_(1, 44.53)_ = 78.89; *p* < .000), Oral Control (*F*_(1, 48.80)_ = 14.31; *p* < .000), and orthorexic behaviours (*F*_(1, 48.06)_ = 10.89; *p* < .001). The first class includes ED patients who are not anxious about fattening, who rate their own weight as “average”, with an EAT-26 score below the cutoff, and frequent engagement in orthorexic behaviours. On the other hand, the second class includes ED patients who are anxious about fattening, rate themselves as overweight, report high levels of ED pathology, as suggested by the EAT score, and engage less frequently in orthorexic behaviours. Moreover, ED patients in the first class evaluated their health significantly higher than those in the second class. A detailed description of the characteristics of both classes is presented in Table 3.

**Table 3:** Characteristics of distinguished classes of eating disorder patients

	Class 1 (*N* = 31)	Class 2 (*N* = 20)
Actual weight	58.53 (*SD* = 10.29)	56.67 (*SD* = 9.38)
Body mass index	21.45 (*SD* = 3.91)	20.34 (*SD* = 2.89)
Ideal weight	48.58 (*SD* = 6.79)	52.66 (*SD* = 6.14)
Highest weight	65.63 (*SD* = 19.01)	59.93 (*SD* = 10.98)
Lowest weight	45.08 (*SD* = 9.18)	44.21 (*SD* = 6.27)

### Predictors of orthorexia nervosa

We investigated whether the EAT-26 and MBSRQ scores could predict orthorexia nervosa. In the SEM model (Table 4) we introduced two new latent variables created from observed averaged scales: the first was measured by Self-classified Weight and Overweight Preoccupation scales, which capture aspects related to self-appraisals of one’s weight; the second latent variable was measured by Dieting and Bulimia and food preoccupation, which captured the most important features of ED pathology.

**Table 4:** Structural equation model prediction of orthorexia nervosa in patients with eating disorders

	Is regressed on	Estimate	*p*
Dieting	Eating pathology	.97	.000
Bulimia and food preoccupation	Eating pathology	.69	.000
Self-classified weight	Weight concern	.63	.000
Overweight Preoccupation	Weight concern	.75	.000
Eating pathology	Orthorexia	−.55	.000
Weight concern	Orthorexia	−.53	.000
Appearance evaluation + body areas satisfaction	Orthorexia	.22	.088
Fitness evaluation	Orthorexia	−.08	.576
Health evaluation	Orthorexia	−.20	.153
Appearance orientation	Orthorexia	−.26	.021
Fitness orientation	Orthorexia	.08	.532
Health orientation	Orthorexia	−.30	.003
Oral control	Orthorexia	−.23	.062

All pathways are standardized regressions

**Fig. 2:**
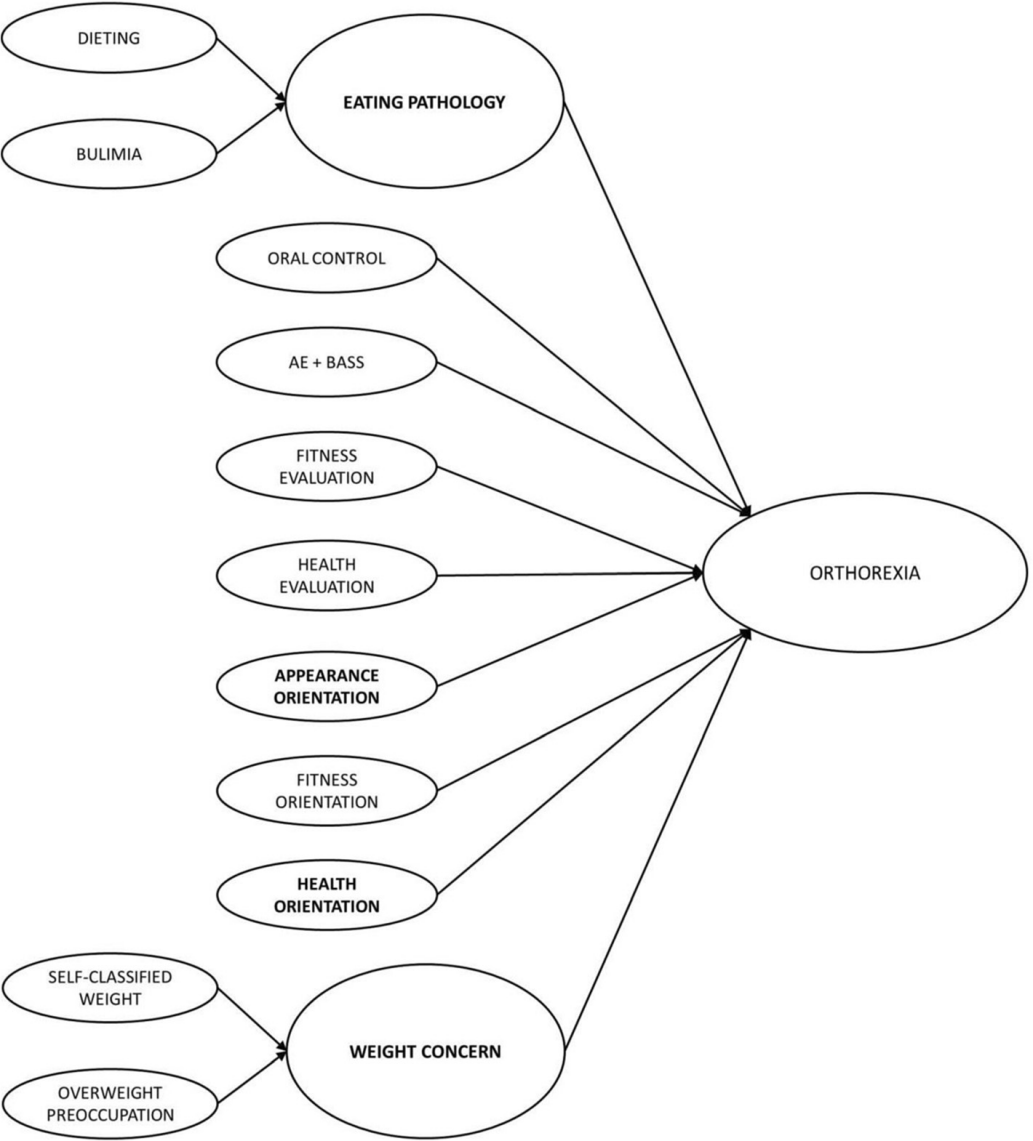
Graphic representation of the Structural Equation Model prediction of orthorexia nervosa in patients with eating disorders. Note. To enhance readability of the Fig. 2, the correlation paths between variables were omitted. Significant predictors of Orthorexia were bolded. AE + BASS = Appearance Evaluation + Body Areas Satisfaction Scale

This SEM model was excellently fitted to the data (*χ*^*2*^_(17)_ = 23.05; *p* = .148; CFI = .962; RMSEA = .08; *p* = .25; SRMR = .05). The graphic presentation of the SEM model is presented in Fig. 2.

Orthorexia nervosa was negatively predicted by eating pathology, weight concern, health orientation and appearance orientation. Pathways between other variables and orthorexic behaviours were not significant. Correlations between variables were moderate, with the exception of a very high correlation between distinguished latent variables (Eating pathology – measured by dieting and bulimia - and Weight concern – measured by self-classified weight and overweight preoccupation; *r* = .85; *p* < .000).

## Discussion

In this study we assessed two important research issues regarding orthorexia nervosa in ED patients. First, we assessed the possibility to identify latent classes in our ED sample; on the basis of the statistical criteria, we were able to distinguish two different latent classes of ED patients. The main differences between latent classes concerned fat anxiety, weight perception and orthorexic behaviours. In the group which reported a lower level of eating pathology, orthorexic behaviours were more frequent; on the contrary, in the group reporting higher levels of eating pathology, orthorexic behaviours were less frequent. These findings are consistent with those by McInerney-Ernst [21], who found that in a group of American female students, orthorexic symptoms were related to a lower level of eating pathology, while less orthorexic traits were found in students with symptoms strongly associated with severe EDs. Eating-related disturbances have been suggested as risk factors for orthorexia [2], and the reverse is also possible. Indeed, as underscored by Mac Evilly [22], orthorexia may be a risk factor predicting a future ED. Orthorexic eating habits could become more and more restrictive and compulsive, actually resembling ED symptoms [22]. Moreover, studying the relationship between orthorexia and EDs, Segura-Garcia and coworkers [7] found that orthorexia may precede the onset of a full-syndrome ED, or represent its evolution during remission and recovery. Similarly, according to Cartwright [23], orthorexia could precede or follow anorexia, and orthorexic behaviour could be a socially-approved way to express anorexic symptoms. What described by Segura-Garcia [7] and Cartwright [23] is likely to impact on patients’ score on questionnaires assessing ED symptomatology and orthorexia. Specifically, we may suppose that patients with an acute, full-syndrome ED may score higher on the first and lower on the latter; on the contrary, patients in an early or late stage of their ED may report less ED symptoms and display more orthorexic features. Anyway, these issues are still controversial; the current literature is not univocal and it is not clear whether orthorexia is a new ED, a different psychiatric disorder, a disorder at all, or a variant of the existing EDs.

As far as our results are concerned, we were surprised finding a group of clinically-diagnosed ED patients without the typical ED symptoms as assessed with the EAT. Nonetheless, some hypotheses can be suggested. First, as described in the results section and in Table 1, it should be noted that in our sample most patients suffered from bulimia nervosa, with a relatively brief duration of illness, and these clinical features may reflect themselves in the questionnaire scores. We should also consider that the EAT is a screening, self-report instrument, with all the limitations that this entails, including the impossibility to exclude dissimulative behavior and hiding the symptoms on behalf of patients. ED patients could show this attitude towards the psychological test in the attempt to adapt themselves to the existing social norms, to hide the real disorders, or perhaps, according to the belief that doing so would shorten their treatment.

We can suggest another hypothesis, considering that in our sample ED patients scoring low on the EAT scored high on the ORTO-15, and that we found a negative correlation between orthorexia and health orientation. It is possible that some patients score low on the EAT but high on the ORTO-15 because they “mistake” their symptoms for healthy behaviors or try to convince themselves (or others) that their behaviors are healthy, and classify them according to this distorted assumption. The result is that ED symptoms may be denied or hidden under a healthy facade. Actually, patients in the first class we identified (low scores on the EAT, high scores on the ORTO-15) evaluated their health higher than patients in the second class.

Anyway from a clinical standpoint, whatever the relationship between orthorexia and EDs, the overlaps between orthorexia and EDs, as well as their differences, should be considered when developing a therapeutic program tailored to the patients’ specific needs. For instance, as already described, orthorexic individuals and ED patients focus on quality/purity of food and quantity of food, respectively. The distorted cognitions underlying an ED are different from those typical of orthorexia nervosa, and should be differently addressed.

The second aim of the study was the identification of predictors of orthorexia nervosa in the ED patients group, which was assessed using the SEM methodology. In SEM we are testing complex regression models, therefore the stronger the coefficient is, the more the independent variable is predicted by the dependent one. All of significant predictors were negatively explaining orthorexic behaviours. This supports the findings described above, from the LCA, i.e., that orthorexic behaviours in the clinical group we assessed are more frequent in those patients with a less severe ED pathology, less weight concern and appearance orientation. The strongest (negative) predictors of orthorexic behaviours were ED pathology and weight concern, with both predictors highly correlated. Anyway these results should be interpreted with caution, considering the small sample size and the fact that ED pathology and weight concern share issues such as fat anxiety, dieting, eating restraint or food and weight perception. As suggested by the LCA, only behaviours related to ED pathology are negative predictors of orthorexia nervosa.

On the other hand, unexpectedly, health orientation, which measures the extent of investment in healthy lifestyle and appearance orientation, which measures the extent of investment in own appearance emerged as a negative predictors of orthorexia. This may be surprising considering that orthorexia has been defined by Bratman & Knight [24] as “a fixation on eating healthy food” in order to avoid ill health and disease. An hypothesis is that a discrepancy might exist between healthy lifestyle as conceived by orthorexic individuals and what is a real and actual healthy lifestyle. As discussed above, since we included patients with a clinical diagnosis of ED, maybe in our sample the surface is orthorexia, but the underlying reason for this behavior is an eating disordered attitude, which has little or nothing to do with avoidance of ill health and disease. The concept of healthism as described by Haman and coworkers [25] may be helpful clarifying this point. Healthism refers to a social construction of health, a “new health consciousness” assuming that health can be achieved easily through individual discipline and moral conduct, focusing on body size and shape [26, 27]. This point of view may not coincide with actual health. Indeed, healthism practices can lead to an improvement of one’s habits as well as to unhealthy behaviors and ideas about body shape, diet and physical exercise [25, 27]. The criteria for what is considered healthy are likely to be highly personal [28]. In the healthism model, the individual is blamed for health problems, and social pressures exist towards constrained behaviors of self-surveillance, which may eventually be transformed into unhealthy, harmful and even destructive behaviors [29, 30]. The risk is that detrimental behaviors become normalized, and that ED patients replace in a more socially acceptable way the typical ED symptoms with orthorexic behaviours.

Moreover, based on the literature [6, 31–33], we should consider that, although it seems that orthorexic behaviours have more parallel with anorexia nervosa than with bulimia nervosa, it is noteworthy that the similarities between anorexia nervosa and orthorexia nervosa (e.g., intense anxiety regarding certain foods and their avoidance, need for control, ego-syntonic nature [34]) have not been empirically established [33]. In our study most patients were diagnosed with bulimia nervosa and classified as normal weight. Like bulimia nervosa, orthorexia is a disorder rooted in food intake. Patients with unhealthy fixation on healthy eating are restricting whole food groups. However, dieting in bulimia nervosa is associated with increased food restriction [35]. Unlike bulimia nervosa, orthorexia nervosa is about control of food intake.

## Conclusion

Some limitations should be underscored and might be targeted in future studies. First, since this is a pilot study, the sample size is small and may limit the generalizability of results. Second, no information was gathered about comorbid psychiatric diagnoses. Last, we assessed orthorexia with a self-report measure, which might have caused an overestimation of orthorexia nervosa in our sample. Moreover, since orthorexia is not a diagnosis, its prevalence can not be described, hence we rather stated the percentage of individuals scoring high on the assessment instrument we have used. Nonetheless, these preliminary results add to the paucity of studies about the assessment of orthorexia in ED patients. Briefly, in the current study we have found that the frequency of orthorexic behaviours decreases alongside with the increase of ED pathology. This conclusion is supported by both the methodological approaches used: person-oriented perspective (LCA) and variable-oriented perspective (SEM). A patient-oriented therapeutic approach might benefit of this discrimination.

## Abbreviations

ED: Eating disorder
EAT-26: Eating Attitude Test-26
BMI: Body Mass Index
MBSRQ: Multidimensional Body-Self Relations Questionnaire
LCA: Latent Class Analysis
SEM: Structural Equation Model
CFI: Comparative Fit Index
RMSEA: Root Mean Square Error of Approximation
SRMR: Standardized Root Mean Residuals
